# Elemental and macromolecular modifications in *Triticum aestivum* L. plantlets under different cultivation conditions

**DOI:** 10.1371/journal.pone.0202441

**Published:** 2018-08-28

**Authors:** Maria-Emiliana Fortună, Viorica Vasilache, Maria Ignat, Mihaela Silion, Tudor Vicol, Xenia Patraș, Ionel Miron, Andrei Lobiuc

**Affiliations:** 1 “Petru Poni” Institute of Macromolecular Chemistry, Iasi, Romania; 2 “Alexandru Ioan Cuza” University, Interdisciplinary Research Department—Field Science, Iasi, Romania; 3 Alexandru Ioan Cuza University of Iaşi, Faculty of Chemistry, Iaşi, Romania; 4 "Apollonia" University, Iasi, Romania; 5 Alexandru Ioan Cuza University of Iaşi, Faculty of Biology, Iaşi, Romania; 6 CERNESIM/L2, Alexandru Ioan Cuza University of Iaşi, Iaşi, Romania; 7 ”Stefan cel Mare” University, Faculty of Food Engineering, Suceava, Romania; College of Agricultural Sciences, UNITED STATES

## Abstract

Young wheat plantlets (wheatgrass), represent a significant source of minerals, enzymes, vitamins, while also rich in phenolics and chlorophylls, with considerable bioactivities. As the biosynthesis of such compounds may be influenced by growth conditions, the current research assesses wheatgrass composition in soil based and hydroponic systems, using water with different elemental composition. FTIR spectroscopy did not reveal significant variations between juice and extracts cultivated in different setups. Surface elemental composition indicated higher Na, P, Si concentrations in hydroponic plants, while AAS analyses showed increased Ca and Mn in soil presence. HPLC-MS of extracts showed that soil and spring water increased chlorophyll and hydroxychlorophyll a concentrations. Phenolic contents were higher in hydroponic plants, while maximum values were recorded for spring water. Radical scavenging activity was stimulated by the use of spring water. Results indicate that wheatgrass with improved mineral and macromolecular composition may be obtained using accessible cultivation setups.

## Introduction

Wheat (*Triticum aestivum* L.) is a staple food cultivated worldwide, supplying carbohydrates, proteins, minerals, vitamins, as well as fibers and antioxidants when conditioned seeds, generally as flour, are used in various products. Seeds are also a good source of antioxidant compounds, such as carotenoids, tocopherols, tocotrienols, phenolic acids, phytic acid, phytosterols and flavonoids [1]. Young wheat plantlets, aged 6–14 days, termed wheatgrass, are also considered an excellent source of bioactive compounds such as vitamins, (A, B, C and E), minerals such as iron, calcium, magnesium, benzo(a)pyrene, ferulic, gallic, caffeic, syringic and p-coumaric acid [2]. The main uses of wheatgrass are the production of wheatgrass juice, which is consumed raw, usually immediately after preparation, but commercially, dried powders or capsules based on wheatgrass also exist. Elevating bioactive contents in the raw material offers the opportunity of developing products (fresh or conditioned) with improved nutritional and therapeutic qualities and the interest in such products is seen in consumer preferences but also in governmental programs such as HarvestPlus, Biofort or HarvestPlus-China, which are operational and targeted at fortifying staple foods such as wheat with, for instance, minerals [3].

Among bioactive substances in wheatgrass, the predominant ones are chlorophylls, up to 18.5 mg/g [4]. Structurally, chlorophyll is composed from a porphyrin (tetrapyrrole) ring, esterified to a phytol, holding a Mg atom in the centre, thus resembling heme in hemoglobin. Chlorophyll derivatives such as pheophytin, metal-pheophytin or metal-chlorophyllin occur as catabolism products in plants, but themselves having significant biological activity. Chlorophylls display antiinflammatory and antiproliferative activities, being able to induce synthesis of Phase II enzymes or inhibition of P450 cytochrome family enzymes, as antiproliferative mechanisms and even minimal intake levels by humans may prove beneficial [5]. As a result, wheatgrass is regarded as a potent medicine, with significant antioxidant activity, which can be used as an adjuvant in the treatment of conditions such as thalassemia [6]. It may help in regulating blood pressure and glycemia [7], has antimicrobial activity and it also contains immunologically active oligosaccharides, specifically maltoheptaose, that may regulate cytokine expression in human peripheral blood mononuclear cells [8] and it also reduces total cholesterol, low density lipoproteins and triacylglycerols [9].

Considering that wheatgrass reflects actually plants at the onset of their development, their chemical composition can be influenced by various factors, among which water availability and composition, fertilization regimes [10] or light quality [11]. Supplementation with N, P, K in the growth medium leads to increased carbohydrate, carotenoid and protein contents in wheatgrass but it may also reduce the contents of phenolic compounds or several minerals [12]. Soil presence in the cultivation substrate, as compared to hydroponic cultivation, may increase the levels of some mineral elements, such as K or Mn in wheatgrass [13]. However, previous studies did not include detailed analysis of one of the major constituents of wheatgrass plants and juice, chlorophyll pigments and derivatives, molecules with significant biological activity. Furthermore, in other researches, elemental composition and macromolecular analysis were basically addressed, while they are useful for a thorough caharcterisation of a product with significant therapeutic potential.

Considering the significant therapeutic applications of wheatgrass, which appear related to its chemical composition, the use of different substrate compositions and cultivation conditions (soil based or hydroponic) may represent viable techniques for obtaining higher quality wheatgrass, possibly tailored to certain requirements such as mineral fortified food products, antioxidant supplements or food grade extracts.

As such, the present paper sets as objectives the assessment of the influence of two types of cultivation methods–soil based and hydroponic–and of two types of water with different elemental composition–spring water and drilling water–on: a) the elemental composition, b) the chlorophyll and chlorophyll derivatives levels and c) phenolic contents and antioxidant levels in wheatgrass, hypothesizing that a variability in mineral levels and cultivation substrate type will lead to variability in wheatgrass quality (described by above mentioned parameters), allowing for selection of cultivation conditions.

## Materials and methods

### Cultivation conditions

Wheatgrass plantlets were produced from wheat seeds–*Triticum aestivum* L.–obtained from a local source. Seeds were placed on 30x50 cm plastic trays, either directly for hydroponic cultivation, or on a layer of soil obtained from a beech forest. Trays were drip watered daily in 2 sessions of 15 minutes each at 12 hours intervals. Two types of water were used, one from a 70 m water drilling and the other from a natural spring, both from locations in Neamt county, Romania. The 4 treatment variants consisted of plantlets grown a) on soil or b) in hydroponic conditions, each watered with 1) spring or 2) drilling water. Wheatgrass plantlets were grown for up to 14 days in controlled conditions (temperature and illumination), then they were harvested and conditioned for analyses immediately. For each variant, six trays were used for replication, the sampling being performed thrice per variant, from each two trays. Each sample was analyzed individually and the results were statistically analyzed using IBM SPSS v. 20 software. The cultivation took place between 15.06–29.06.2017, in the laboratories of the Faculty of Biology, „Alexandru Ioan Cuza” University of Iasi, Romania.

### Preparation of wheatgrass extracts

The wheatgrass juice was obtained by cold extraction using a commercial extractor. Extraction of chlorophyll type pigments from wheatgrass were performed by maceration using an extraction mixture composed of methanol and petroleum ether in a 2:1 ratio. Maceration was performed for 24 h avoiding direct light then the extracts were filtered and used for column chromatography separation [14]. Column chromatography used silica gel as the adsorbent (silica gel 60, Merck) and n-hexane/acetone mixture in a gradient from 0:1 to 1:0 ratios (v/v).

### Elemental composition assessment

The concentration of the elements was measured from solutions by flame atomic absorbtion spectroscopy (FAAS). The data were acquired using a Continuum Source Atomic Absorption Spectrometer—contrAA 300—equipped with an optimized high-resolution Echelle double monochromator.

### Wheatgrass FTIR characterization

FTIR (Fourier Transform Infra Red) absorption spectra of wheatgrass juice and fractions of chlorophyll extracts were recorded using a Bruker Vertex 70 FTIR spectrometer, performed in transmission mode within 400–4000 cm^-1^ range with a resolution of 2 cm^-1^ at room temperature on samples dissolved in KBr pellets.

### HPLC-MS analysis of chlorophyll and derivatives

Chlorophyll and chlorophyll derivatives of wheatgrass were assessed by HPLC-MS in successive fractions obtained by column chromatography. The HPLC-MS analyses were carried out using an Agilent 6500 Series Accurate-Mass Quadrupole Time-of-Flight (Q-TOF) LC/MS system, equipped with a binary pump, heated column compartment, automatic injection system (autosampler) and diode array detector (UV-VIS DAD). The optimal conditions for separations were achieved using an a Zorbax SB C18 reverse phase column (4.6 mm x 150 mm, 5 μm particle size) with a column temperature kept at 40°C, using a 50 μL injection volum, solvent flow 1 mL/min, elution in gradient: 10% B at 0 min; 40% B at 3 min; 50% B at 5 min, came-back to 10% B in 10 min and column equilibration in 10 min, where (A) is methanol and (B) acetonitrile. The separation process was monitored by UV-VIS DAD detector at 430 and 660 nm. The ESI-Q/TOF MS conditions were set as follows: electrospray ionization (positive ion mode), drying gas (N2) flow rate 7.0 L/min; drying gas temperature 325°C; nebulizer pressure 30 psi, capillary voltage 4000 V; fragmentation voltage 200 V; the full-scan mass spectra of the investigated compounds were acquired in the range m/z 50–3000.

### Phenolic content assays

Total phenolic and flavonoid contents were assayed from wheatgrass juice, diluted as needed, as described in [15]. Briefly, for total phenolics, aliquots of wheatgrass were mixed with Folin reagent for 5 minutes, then 7.5% Na_2_CO_3_ was added and absorbance of solutions was read at 760 nm after 90 minutes. For flavonoids, wheatgrass juice (0.15 ml) was mixed with NaNO_2_, incubated for 5 minutes, followed by addition of AlCl_3_ and further 6 minutes incubation, then 1M NaOH was added and absorbances were read at 510 nm. Results were expressed based on calibration curves using gallic acid and, respectively, quercetine.

### Free radical scavenging activity assay

Free radical scavenging capacity of wheatgrass juice was assessed using the DPPH free radical method, as performed in [15]. Briefly, 2.9 ml of a 40 μM DPPH was incubated with 0.1 ml of wheatgrass juice for 90 minutes and absorbance was read at 515 nm. Results were expressed as % decolorisation compared to the original DPPH solution.

### Statistical analyses

All differences between means were assessed using ANOVA followed by Tukey post-hoc tests, at a significance level of p<0.05. Calculations were performed using XLStat 2018.3 from Addinsoft.

## Results

### Elemental composition

The elemental analysis of the two types of water used for wheatgrass irrigation revealed differences among their composition (Table 1). Spring water had more Ca, Fe, K and Mg than drilling water which, in turn recorded more Na, with significant differences for all elements, except iron.

**Table 1:** Elemental composition of wheatgrass juice and of waters used for wheatgrass irrigation.

Elements	Ca	Fe	K	Mg	Mn	Na
Water (mg/l)						
Drilling water	7.745^a^±0.116	0.0438^a^±0.01	1.117^a^±0.013	12.28^a^±0.101	-	6.094^a^±0.002
Spring water	27.42^b^±0.296	0.0532^a^±0.008	3.448^b^±0.014	43.46^b^±0.606	-	3.462^b^±0.047
Wheatgrass juice (mg/100 ml)						
Hydroponic/Drilling water	117.22^a^±0.061	0.27^a^±0.002	74.05^a^±0.008	8.44^a^±0.129	3.35^a^±0.003	78.88^a^±0.019
Hydroponic/Spring water	126.40^b^±0.042	0.29^b^±0.002	75.00^a,b^±0.027	12.02^b^±0.271	3.19^b^±0.001	69.27^b^±0.028
Soil/Drilling water	157.55^c^±0.140	0.33^c^±0.004	44.59^c^±2.184	5.61^c^±0.037	10.19^c^±0.002	75.28^c^±0.018
Soil/Spring water	208.15^d^±0.126	0.28^a,b^±0.001	92.90^d^±0.016	14.59^d^±0.158	9.56^d^±0.006	55.19^d^±0.090

Different letters in the same row indicate significantly different means for p<0.05

Regarding the biological material, surface elemental analysis indicated variations in mineral concentration in wheatgrass leaves, according to cultivation conditions (Table 2). In plantlets grown on drilling water, Na, Cl and K had higher values, with up to 162.5%, 86.2% and, respectively, 23.7% compared to spring water grown plantlets, significant differences being recorded between drilling and spring water cultivation for Na and K. Hydroponically grown plants showed significantly higher concentrations of P, with 44.6% and S, with 106.2% compared to plants grown in soil. Other elements assessed with this technique did not show significant differences among treatments.

**Table 2:** EDX elemental composition of wheatgrass leaves.

Element, Wt %	Hydroponic/Drilling	Hydroponic/Spring	Soil/Drilling	Soil/Spring
C	51.71^a^±0.38	54.27^b^±0.36	56.96^c^±0.54	54.65^b,d^±0.54
N	6.95^a^±0.52	7.08^a^±0.25	7.14^a^±0.36	7^a^±0.47
O	33.08^a^±0.22	32.14^a,b^±0.51	28.59^c^±0.47	32.31^a,d^±0.43
Na	0.84^a^±0.04	0.32^b^±0.04	0.52^c^±0.03	0.32^b,d^±0.05
Mg	0.45^a^±0.07	0.4^a^±0.03	0.48^a^±0.05	0.44^a^±0.05
Si	0.15^a^±0.06	0.14^a^±0.03	0.28^a^±0.04	0.16^a^±0.04
P	1.62^a^±0.04	1.32^b^±0.04	1.13^b,c^±0.04	1.12^c,d^±0.05
S	0.99^a^±0.15	0.75^a,b^±0.03	0.48^b,c^±0.06	0.49^b,c,d^±0.04
Cl	0.46^a^±0.06	0.29^a^±0.05	0.54^a^±0.07	0.33^a^±0.05
K	3.34^a^±0.05	2.75^b^±0.03	3.33^a,c^±0.08	2.7^b,d^±0.03
Ca	0.39^a^±0.05	0.54^a^±0.07	0.57^a^±0.04	0.45^a^±0.05

Different letters in the same row indicate significantly different means for p<0.05

Calcium, natrium and potassium in juice followed the levels of these two elements in the irrigation water. Specifically, when plantlets were grown on soil using spring water, significant 2.54 fold higher Ca, 2.08 fold higher K and 2.53 fold higher Mg contents were recorded compared to drilling water grown plants. When hydroponic cultivation was employed, the differences in mineral levels between variants were smaller.

In the same time, the use of drilling water favoured the uptake of Na in wheatgrass plants, corresponding to the highest Na amounts in the two types of water used, also confirming EDX data.

### FT-IR analysis

**Fig 1:**
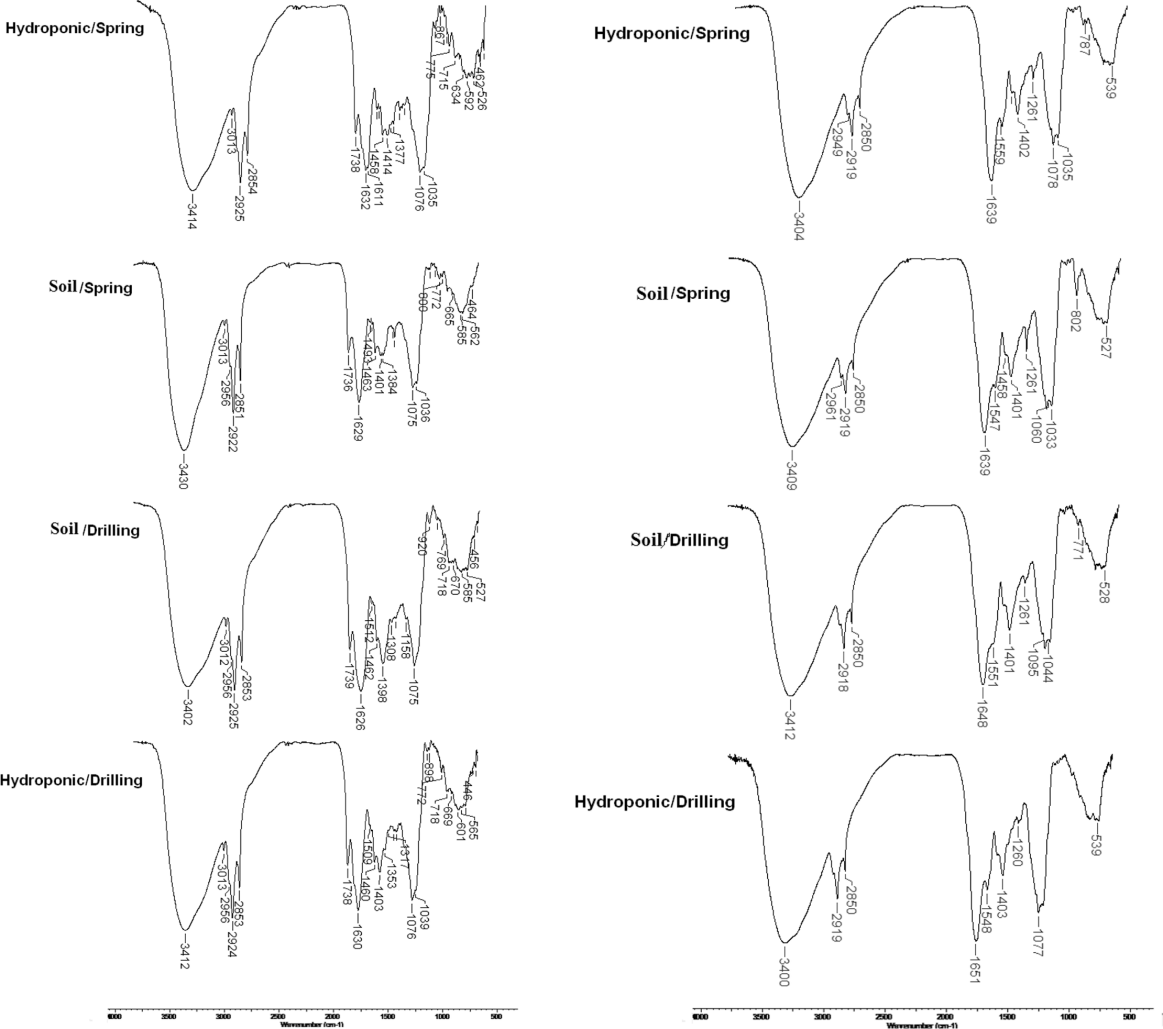
FT-IR spectra of wheatgrass juice (left) and wheatgrass extracts (right).

The FT-IR spectra of wheatgrass methanol:petroleum ether extracts and wheatgrass juice, corresponding to plantlets cultivated in soil and hydroponic substrates grown on drilling water and spring water are shown in Fig 1. In both types of samples, characteristic bands were observed around 1736–1739 cm^-1^, corresponding to ester C = O groups.

The peaks around 1630 cm^-1^ may correspond to C = C bonds in chlorophyll molecules, while the broad peaks around 3400 cm^-1^ represent–OH groups of alcohols. Peaks at approximately 1560 cm^-1^ are characteristic to N-O links and the peaks at 1075 cm^-1^ relate to C-O of alcohols and phenols. The peaks at 1458–1462 cm^-1^ observed in wheatgrass extracts may be an indicative of the Mg porphyrin nucleus in the chlorophyll molecule [16]. No major differences were recorded among FTIR spectra of the wheat plantlets grown in the four experimental setups.

### Assimilatory pigment HPLC-MS analysis

**Fig 2:**
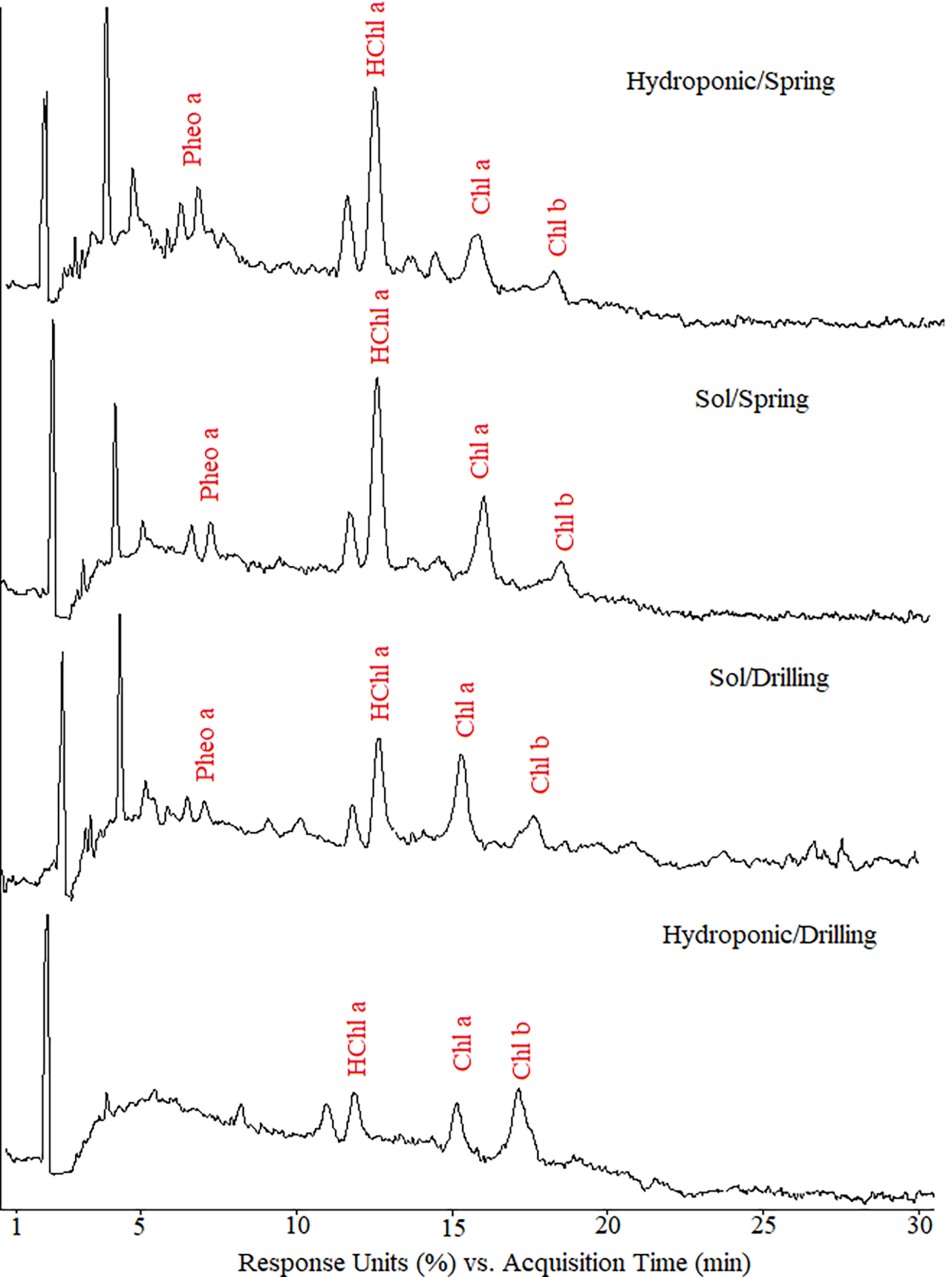
HPLC chromatograms (430 nm) of chlorophyll extracts of wheatgrass cultivated in different systems.

**Fig 3:**
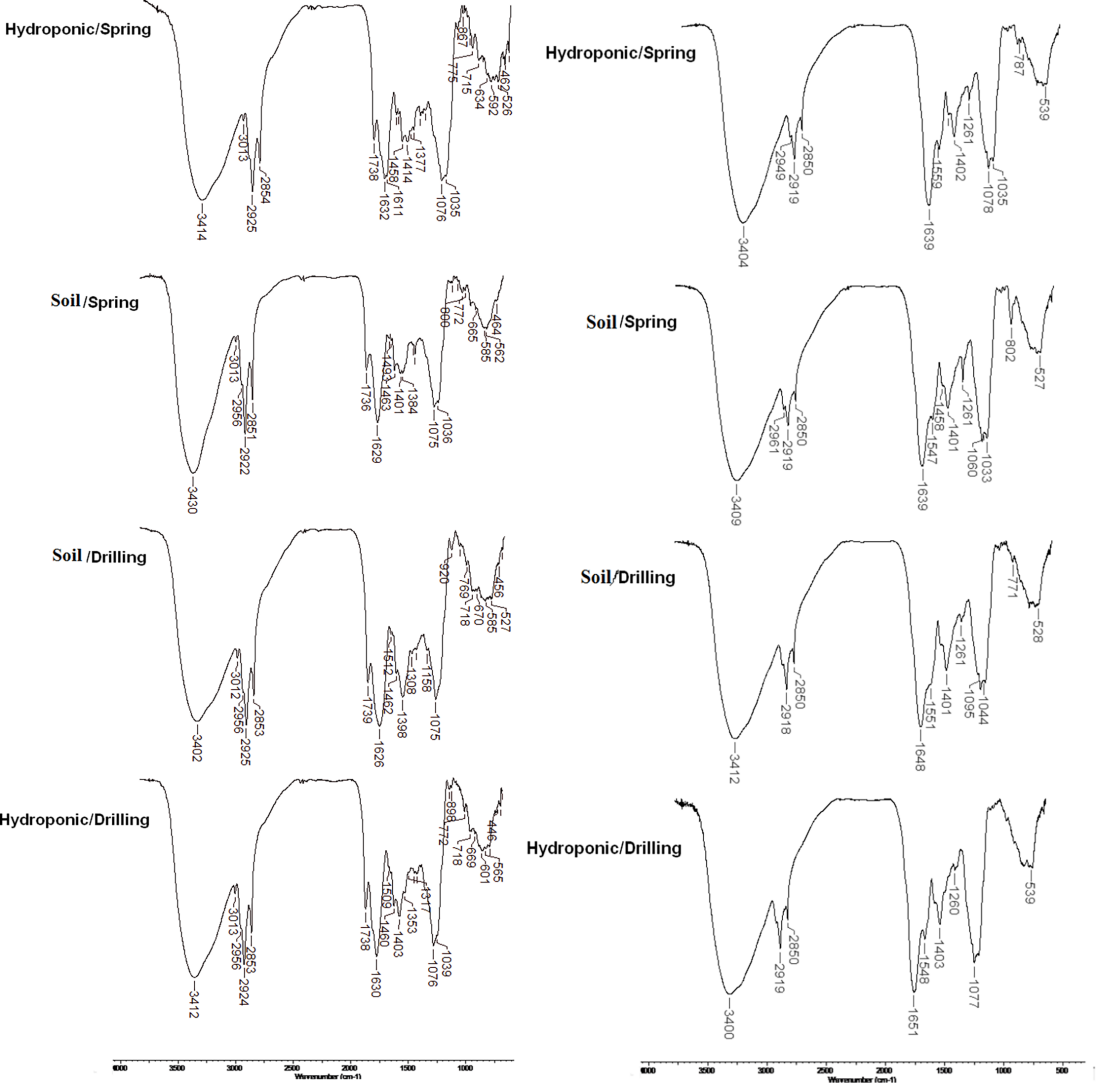
(+) ESI-MS spectra of identified pigments in wheatgrass extracts.

The chromatograms recorded at 430 nm (Fig 2) show the presence of several pigments, four of which were identified by ESI-MS analysis: pheophytin a (6.1 min), hydroxychlorophyll a (13.1 min), chlorophyll a (15.5 min) and chlorophyll b (16.8 min). The ESI-MS spectra of the identified pigments are shown in Fig 3. For pheophytin a, hydroxychlorophyll a, chlorophyll a and b, the base peak was observed at m/z 871.68, 909.52, 893.53 and respectively, 907.74 which corresponds to protonated form [M+H]^+^. In addition, ion corresponding to the single charge sodium adduct [M+Na]^+^ were observed at *m/z* 931.53 for hydroxychlorophyll a.

Quantitatively, plants hydroponically grown on spring water recorded elevated amounts of pheophytin, chlorophyll a and hydroxychlorophyll a, compared to drilling water, as established by HPLC-MS analysis (Table 3). Comparing the type of substrate, higher contents of chlorophyll a were recorded when soil was used. Spring water led to increased chl a contents in both soil and hydroponic substrates, with larger differences under hydroponic cultivation. Chlorophyll b contents appeared to vary without respect to the substrate or type of water, with higher values in hydroponic/drilling and soil/spring plantlets. All differences recorded were significant from a statistical point of view.

**Table 3:** Pigment content in wheatgrass leaves extracts.

Pigment content (mg/g)	Hydroponic/Spring	Soil/Spring	Hydroponic/Drilling	Soil/Drilling
Phaeophytin	23.58^a^±0.29	4.31^b^±0.29	9.42^c^±0.04	7.41^d^±0.06
Hydroxychlorophyll a	65.1^a^±0.18	38.52^b^±0.41	62.09^c^±0.11	24.4^d^±0.22
Chlorophyll a	52.01^a^±0.26	40.42^b^±0.29	30.17^c^±0.22	25.84^d^±0.09

Different letters in the same row indicate significantly different means for p<0.05

### Phenolic contents and free radical scavenging of wheatgrass juice

Phenolic contents and free radical scavenging activities were influenced up to a certain extent by cultivation conditions (Table 4). With the same type of water, both total phenolics and flavonoids were higher in hydroponic grown plants compared to soil grown ones, although only flavonoids in wheatgrass cultivated with spring water displayed significant differences between cultivation systems. Considering the influence of water composition, spring water led to elevated levels of phenolic compounds compared to drilling water, with the most notable difference in the case of flavonoids. Free radical scavenging activity appeared to be influenced more by the type of cultivation, once again with hydroponic system leading to increased activity, but no significant differences were recorded. However, in soil system, spring water induced higher scavenging activity.

**Table 4:** Phenolic contents and free radical scavenging activities of wheatgrass juice.

Cultivation condition	Hydroponic—spring	Soil—spring	Hydroponic—drilling	Soil—drilling
Total phenolic content (mg gallic acid/ml juice)	818.9^a^±4.93	780.23^a,b^±2.6	746.57^b,c^±19.62	706.93^c,d^±20
Total flavonoid content (mg quercetin/ml juice)	809.71^a^±68.01	490.67^b,c,d^±14.1	467.33^b,c,d^±23.71	346.86^b,c,d^±9.51
Free radical (DPPH) % scavenging	48.93^a^±2.06	38.31^a^±7.97	48.93^a^±3.8	30.19^a^±2.84

Different letters in the same row indicate significantly different means for p<0.05

## Discussion

### Cultivation conditions effects on elemental composition of wheatgrass

In previous reports, the contents of Ca and Mg of wheatgrass were higher in plants grown only on water or nutrient solution compared to plants grown on soil [13], while K, Mn, Zn, Fe, Na were higher in soil grown wheatgrass compared to plantlets grown only on water [16]. The main difference between soil and hydroponic cultivation with regard to mineral nutrition relates to the influence of soil matrix, as the cation exchange complex and surface related parameters of soil may alter mineral availability. Also, plant-water relations are also influenced as, in soil systems, not only osmotically active substances determine water uptake but also the matrix potential [17].

Nitrogen is one of the most influential macroelement in plant physiology and development, however, N concentrations in wheatgrass plantlets were not different among treatments. This, as wheat is known to be nitrogen-sensitive and nitrogen uptake directly correlates with nitrogen availability, indicates that the cultivation system had little effect on the uptake of this element, possibly due to fulfillment of nitrogen requirements of wheat by the substrate in all variants.

Manganese is a micronutrient that is less an integral part of enzymes than Cu, Zn or Fe are, rather it acts as an enzyme activator, examples being pyruvate oxidase, NAD kinase, pyruvate kinase, PEP carboxylase, hexokinase etc., although also found in the constituion of some enzymes such as Mn-protein in photosystem II (PSII) and the Mn-superoxiddismutase. Meanwhile, Mn is essentially related to photosynthesis, with involvment in oxygen-evolving reactions through water splitting, as well as in the structural edification of the chloroplast lamellae [18]. The higher uptake of Mn in soil grown wheat compared to hydroponic one is clearly a result of Mn being absent (below detection limits) in irrigation water alone, but supplied by soil when this cultivation system was used. This is sustained by the fact that manganese is initially rapidly taken up by roots, especially as Mn^2+^ and is mobile in plants. Via xylem pathways, it is being quickly translocated, accumulating predominantly in shoots rather than roots, when present in sufficient concentrations in substrate [19]. The patterns of Mn accumulation in wheatgrass suggest a possible positive interaction with Ca and a negative one with P, the latter being able to precipitate Mn within roots. Potassium helps in regulation of pH and osmotic levels, as it does with stomatal opening and closing and, therefore, it plays a significant role in the water status of the plants. It is also an important elements in cell extension, protein synthesis and photosynthesis [20]. The results indicate that soil potassium supplies were adequate, and apparently, no leaching occured during irrigation as potassium is rather immobile in soil. However, in hydroponic setup, where no potassium baseline existed, the uptake was greatly influenced by the concentration of this element in the irrigation water. Potassium concentration in the nutrient solution correlates directly with K levels and is inversely correlated with Mn in plant tissues, decreasing the latter by reducing root absorption rates [21].

Natrium is a non-essential nutrient, however low Na concentrations appear to stimulate plant growth. In wheatgrass, the inverse trend of Na uptake with several elements, most notably K, Mg and Ca, occurs as a result of the fact that these elements have similar chemical attributes. As such, Na can replace K and Ca in several physiological functions, such as photosynthesis, nutrition, growth, water and ion transport, thereby reducing the need and uptake for these minerals [22].

When considering differences in mineral uptake, although element levels in the solution are main factors affecting mineral uptake, energy demand of plants for transport (passive or active) and element characteristics must be accounted for. The more marked influence of the type of cultivation system on Ca, Mn, P or S uptake by wheatgrass relates to their possible divalent character in the substrate, which makes these minerals less readily absorbed by roots, but more accessible in hydroponic cultivation. Other elements, such as monovalent K and Na are more easily available and the amounts in the substrate, that is, the type of water used, play a more important role in their uptake and accumulation.

Considering the high amounts of minerals in wheatgrass, it may be regarded as a potential source of such elements, a source that may be subjected to fortification with respect to certain minerals. Humans need at least 22 minerals in their diet for optimum physiological functions, however it is estimated that the world population has a diet deficient in Fe (60%), Zn (30%), Ca, Mg, etc. Therefore, biofortification of raw materials for food such as plants is an effective way to address these deficiencies and it may be achieved by supplying optimum amounts of minerals in the cultivation substrate [23]. Minerals such as N, K, P, Ca, Mg, S, Cl, are essential for humans. Ca deficiency may lead to rickets or eclampsia, is regarded as a cofactor in enzyme reactions (fatty acid oxidation, mitochondrial carrier for ATP) and is involved in the maintenance of the mineral homeostasis [24]. Ca requirements may be fulfilled by increasing Ca intake from food [25], recommended Ca daily intakes being between 300 mg for infants and 1300 mg for adolescents and adults. Magnesium is a regulator of smooth muscle functioning, a cofactor of 300 enzymes and increased intake may prevent diabetes, hypertension and other vascular related conditions [24]. Typical intakes should be between 220–260 mg/day for adults. Sodium plays a role in maintaining electrolyte and osmotic balances, in nerve conduction, active cellular transport and formation of bone apatite while recommended daily allowance is 500–750 mg. Potassium is also a determinant of electrolyte and osmotic balances and also plays a part in assisting nerve transmission and heart contraction, having a recommendation of 3500 mg/day [24]. Iron is an essential mineral especially by its involvement in the structure of hemoglobin and also of enzymes such as cytochromes and is an important part of the electron transport chain and recommended intakes are between 8–18 mg/day [25]. Mn is a cofactor for enzymes such as superoxide dismutase or arginase and an activator for other enzymes while requirements are around 2 mg/day [24, 25].

Wheatgrass juice consumption may contribute towards meeting daily mineral requirements, with 100 ml adding 5–7% of the necessary Mg, 10% of the required Na and more than 60% of the required Ca.

### Modifications of macromolecular composition by nutrient levels

A higher content of certain elements in plant extracts may be positively correlated with higher levels of bioactivities. For example, the amount of elements such as K, Zn and Mg were found correlated with higher values of the antioxidant activity of extracts of wheatgrass [26], one possible explanation for Mg being its presence in the chlorophyll molecule, a compound with significant antioxidant activity. This relation becomes important considering that the antioxidant activity of wheatgrass may be increased with different cultivation conditions, specifically by varying the amounts and the availability of mineral elements in the substrate. [27] proved that wheatgrass grown on soil had higher antioxidant activity in the ABTS, DPPH, ORAC and lipid peroxidation antioxidant assays compared to wheatgrass grown on tap water or nutrient solution.

Differences in chlorophyll contents may be attributed to different cultivation substrates and systems, considering that nutrients such as N and P concentrations are known to influence wheatgrass chlorophyll content, while hydroponic cultivation influences the accumulation of Ca, Cu, Mg and Mn [28]. Similarly, wheatgrass mineral composition and antioxidant activity may be modified by different types of water used for irrigation [12, 27]. Several chlorophyll derivatives occur in plants, either without a metal atom (chlorins, pheophytins, and pyropheophytins) or with one in the center (Mg-chlorophylls, Zn-pheophytins, Zn-pyropheophytins, Cu-pheophytin, Cu-chlorophyllins), the latter with higher antioxidant activity than the former [28]. There are various conditions, including stress or aging, that may cause chlorophyll to degrade into pheophytin (pheo), wich is simply chlorophyll without central Mg-atom. Antioxidant activity is presumed to be owed to the π-cation arrangement of the porphyrin and to the presence of the metal atom as an electron donor, while antimutagenic activity may result from the formation of tight molecular complexes with mutagenic compounds.

Several elements relate to chlorophyll synthesis as part of the chlorophyll molecules (such as N or Mg) or of their synthetic pathway (Fe), participate in enzyme activation (such as Mn), as energy storage (P), in electron transport (Cu) etc. Nitrogen is related to chlorophyll synthesis and N deficiency is known to induce decreases in chlorophyll contents and, consequently, in photosynthetic light reactions and Calvin cycle development [19]. However, N does not appear influenced by cultivation conditions and chlorophyll synthesis seemed to relate to several other elements. The relation between higher chlorophyll contents of wheatgrass and Mg levels in substrate are the result of the fact that 10–20% of the entire Mg amount in leaves are bound to the chlorophyll molecule and is also required for grana stacking. Also, higher concentrations of phaeophytin coupled to lower concentrations of chlorophyll in wheatgrass could relate to chlorophyll catabolism, where Mg is dechelated from chlorophyll molecules and recycled when Mg levels are not adequate [29].

Another element that possibly influenced chlorophyll synthesis in wheatgrass, is K, which is required in certain amounts, especially when Mg is subsufficient, functioning probably as Mg replacer [30]. In the same time, wheatgrass soil grown plantlets recorded 3-fold higher concentrations of Mn compared to water grown plantlets and also displayed higher chlorophyll a concentrations. Mn deficiency may be related to decreased chlorophyll concentrations as well as to modified thylakoid structures, which may explain the relation between chlorophyll contents and Mn in wheatgrass. The requirement of optimum Mn levels in the substrate for adequate chlorophyll synthesis was already proved for tomato plants [17].

However, different species and even different cultivars of the same specie may respond differently with regard to nutrient uptake and to the effect of nutrient availability on bioactive compound levels. As such, higher total phenolic, flavonoid and vitamin C contents and higher antioxidant activity were obtained with unfertilised turfgrass and wheatgrass, while fertilised plants recorded higher Zn and K concentrations, but with variations among cultivars [12]. In hydroponically grown lettuce, Mg and K contents increased qudratically and, respectively, linear, with the concentration of nutrients in the cultivation solution [24], similar with the increase in the same elements in our experiment in relation with the amounts of same elements in the drilling and, respectively, spring water.

Regarding phenolic compounds, minerals such as Mg and Mn are essential for the functioning of some enzymes of the phenylpropanoid and flavonoid biosynthetic pathways (such as phenylalanine ammonium lyase, CoA-ligase or methyltransferases), while deficiencies or excesses of other minerals may increase phenolic levels as an adaptive response. For example, mineral levels influenced significantly the amounts of phenolic compounds in tomatoes and cichory [31]. Hydroponic cultivation led to higher antioxidant activity in basil [32] and to higher phenolic levels in lettuce cv. “*Lollo rosso*” [33]. The decrease in phenolics and antioxidant activity in soil grown wheat may be explained by the fact that increased Mn concentrations in nutrient solution may lead to accumulation of this element in chloroplasts and to reduced chlorophyll synthesis and also to phenolic compounds oxidation and, therefore, to a decrease in total phenolics [34]. In wheatgrass, several factors may influence the amount of phenolic compounds, such as light, temperature and mineral nutrition [9]. In this specie, changes in the production of phenolics and antioxidant activities were observed depending on the characteristics of the cultivation medium (water only or water and soil and the composition of the medium) [22].

The overall results indicate that bioactive compounds in wheatgrass vary accordingly with increased mineral availability. This is somehow in contrast with hypotheses such as Carbon Nutrient Balance or Growth Differentiation Balance, which state that, under sufficient nutrient levels, growth will be favoured over secondary metabolism [35]. However, considering that wheatgrass growth was not limited by the amounts of nutrients in substrate, increases of secondary metabolite synthesis such as phenolics could be the result of stimulation by certain elements. For instance, K+ in higher amounts acts as an enzyme stimulator for pigment synthesis and, being highly mobile, excess is translocated towards phenylpropanoid synthesis, thus stimulating phenolic production [36]. In the mean time, higher doses of Ca and K may lead to stimulation of phenolic synthesis, mainly by upregulating responsible genes [37].

Various biological activities and potential therapeutic uses of wheatgrass juice have been demonstrated or proposed, such as antioxidant, anti-arthritis [3], hepatoprotective, antiproliferative, anti-ulcer. Such activities are generally ascribed to phenolics [2] and to the chlorophyll present in wheatgrass.

Free radical scavenging activity is a highly sought after property in both medicine as well as in nutrition. Free radicals are atoms, molecules or ions with unpaired electrons, that usually accept an electron in order to reach a higher stability [38]. In biological systems, free radicals (such as reactive oxygen species–ROS, reactive nitrogen species–RNS, reactive sulphur species–RSS) may occur from normal metabolic processes, or stresses such as smoking, injuries, pollution etc. Electrons may be donated to these radicals from lipids, DNA, proteins, carbohydrates etc., thus free radicals are associated with aging, inflammatory conditions, artherosclerosis etc. [39].

Among protection mechanisms against free radicals and their deleterious activities, antioxidants such as phenolics (and, specifically, flavonoids) are regarded as a second line of defence, after antioxidant enzymes. The presence of high levels of phenolics, regarded as nutraceuticals and the associated free radical scavenging activity in wheatgrass juice renders it as functional food, considering that such foods provide health benefits beyond basic nutrition. Moreover, the antioxidant activity of wheatgrass is considered to be similar or higher than that of spirulina, a potent and largely used antioxidant [40].

## Conclusions

Wheatgrass plantlets respresent an easy to obtain, rich in biologically active compounds, functional food. Under non-growth limiting conditions, varying the nutrient levels (such as Mg, Ca, K, Mn and Na) and their condition (as only water solved or in a soil matrix), led to wheatgrass with different amounts of minerals, some of them essential for human wellbeing. Wheatgrass with higher concentration of chlorophyll pigments and some of their derivatives, along with higher concentration of phenolics and increased free radical scavenging activity was obtained, hereby confirming the hypothesis that wheatgrass quality may be improved by altering cultivation parameters, in order to obtain superior products.

An important aspect in wheatgrass cultivation is nutrient interactions that may occur and which allow for finer control over wheatgrass composition, however these effects must be investigated at deeper levels, including ranges of concentrations and physico-chemical properties of the substrate, integrated in a multivariate approach. Analyses of enzymatic activity may reveal more precise sites of influence of cultivation factors on secondary metabolite production and such data can be combined with effects from other stimulants of metabolism, such as light or temperature. Moreover, as cultivation parameters such as those investigated in the present paper are facile to alter, wheatgrass improvement is appealing to all types of producers, from home to industrial ones. Wheatgrass, either raw or processed, has a high potential as a functional food, and further investigations should be targeted at establishing optimum nutrient levels and availability for maximum increases in bioactive compounds concentrations and activities, while also taking into account productivity, for economic reasons.

## Supporting information

S1 Table Raw values of analyses of elemental composition of wheatgrass juice and of waters used for wheatgrass irrigation. (DOCX)

S2 Table Raw values of analyses of EDX elemental composition of wheatgrass leaves. (DOCX)

S3 Table Raw values of analyses for pigment content in wheatgrass leaves extracts. (DOCX)

S4 Table Raw values of analyses for phenolic contents and free radical scavenging activities of wheatgrass juice. (DOCX)
