# Supplementary material for: Elemental and macromolecular modifications in Triticum aestivum L. plantlets under different cultivation conditions
Source: PLoS One. 2018 Aug 28;13(8):e0202441. doi: 10.1371/journal.pone.0202441 (PMC6112624; doi:10.1371/journal.pone.0202441)
Supplement: S1 Table — (DOCX) [file pone.0202441.s001.docx]

Table 1. Raw values of analyses of elemental composition of wheatgrass juice and of waters used for wheatgrass irrigation

| **Elements** | **Ca** | **Fe** | **K** | **Mg** | **Mn** | **Na** |
| --- | --- | --- | --- | --- | --- | --- |
| **Wheatgrass juice (mg/kg f.w.)** | | | | | | |
| **Hydroponic/Drilling water** | 117.11 | 0.268 | 74.05 | 8.62 | 3.34 | 78.87 |
|  | 117.19 | 0.275 | 74.03 | 8.51 | 3.35 | 78.86 |
|  | 117.32 | 0.268 | 74.06 | 8.19 | 3.35 | 78.92 |
| Mean ± standard error | 117.21±0.061 | 0.27±0.002 | 74.05±0.0088 | 8.44±0.129 | 3.35±0.003 | 78.88±0.019 |
| **Hydroponic/Spring water** | 126.44 | 0.29 | 74.95 | 12.43 | 3.2 | 69.26 |
|  | 126.32 | 0.295 | 75.04 | 12.13 | 3.19 | 69.27 |
|  | 126.45 | 0.29 | 75.02 | 11.51 | 3.19 | 69.35 |
| Mean ± standard error | 126.4±0.042 | 0.29±0.002 | 75±0.027 | 12.02±0.271 | 3.19±0.003 | 69.29±0.028 |
| **Soil/Drilling water** | 157.305 | 0.325 | 48.03 | 5.67 | 10.19 | 75.18 |
|  | 157.79 | 0.33 | 45.2 | 5.625 | 10.19 | 75.37 |
|  | 157.56 | 0.34 | 40.55 | 5.545 | 10.195 | 75.29 |
| Mean ± standard error | 157.55±0.14 | 0.33±0.004 | 44.59±2.18 | 5.61±0.037 | 10.19±0.002 | 75.28±0.06 |
| **Soil/Spring water** | 207.915 | 0.28 | 92.92 | 14.82 | 9.56 | 55.23 |
|  | 208.345 | 0.275 | 92.91 | 14.67 | 9.55 | 55.015 |
|  | 208.195 | 0.28 | 92.867 | 14.29 | 9.57 | 55.32 |
| Mean ± standard error | 208.15±0.126 | 0.28±0.002 | 92.9±0.016 | 14.59±0.158 | 9.56±0.006 | 55.19±0.09 |
| **Water (mg/l)** | | | | | | |
| **Drilling water** | 7.701 | 0.0667 | 1.104 | 12.38 |  | 6.097 |
|  | 7.96 | 0.0216 | 1.146 | 12.08 |  | 6.093 |
|  | 7.565 | 0.043 | 1.1 | 12.385 |  | 6.092 |
| Mean ± standard error | 7.74±0.116 | 0.0438±0.011 | 1.117±0.0127 | 12.28±0.101 |  | 6.094±0.002 |
| **Spring water** | 27.9 | 0.061 | 3.43 | 42.25 |  | 3.536 |
|  | 26.9 | 0.036 | 3.44 | 44.078 |  | 3.375 |
|  | 27.47 | 0.0599 | 3.475 | 44.06 |  | 3.476 |
| Mean ± standard error | 27.42±0.29 | 0.0523±0.008 | 3.448±0.014 | 43.46±0.606 |  | 3.462±0.047 |
