# Supplementary material for: Elemental and macromolecular modifications in Triticum aestivum L. plantlets under different cultivation conditions
Source: PLoS One. 2018 Aug 28;13(8):e0202441. doi: 10.1371/journal.pone.0202441 (PMC6112624; doi:10.1371/journal.pone.0202441)
Supplement: S2 Table — (DOCX) [file pone.0202441.s002.docx]

Table 2. Raw values of analyses of EDX elemental composition of wheatgrass leaves

| **Element, Wt %** | **Hydroponic/Drilling** | **Hydroponic/Spring** | **Soil/Drilling** | **Soil/Spring** |
| --- | --- | --- | --- | --- |
| C | 50.98 | 53.78 | 57.84 | 53.64 |
|  | 51.89 | 54.97 | 55.97 | 54.83 |
|  | 52.26 | 54.06 | 57.07 | 55.48 |
|  | 51.71±0.38 | 54.27±0.36 | 56.96±0.54 | 54.65±0.54 |
|  |  |  |  |  |
| N | 7.03 | 7.55 | 6.73 | 6.78 |
|  | 7.81 | 6.71 | 7.85 | 6.32 |
|  | 6.01 | 6.98 | 6.84 | 7.9 |
|  | 6.95±0.52 | 7.08±0.25 | 7.14±0.36 | 7±0.47 |
|  |  |  |  |  |
| O | 32.76 | 32.14 | 27.79 | 33.17 |
|  | 32.98 | 31.27 | 28.57 | 31.76 |
|  | 33.5 | 33.02 | 29.41 | 32.01 |
|  | 33.08±0.22 | 32.14±0.51 | 28.59±0.47 | 32.31±0.43 |
|  |  |  |  |  |
| Na | 0.76 | 0.25 | 0.57 | 0.28 |
|  | 0.91 | 0.4 | 0.5 | 0.41 |
|  | 0.86 | 0.31 | 0.49 | 0.27 |
|  | 0.84±0.04 | 0.32±0.04 | 0.52±0.03 | 0.32±0.05 |
|  |  |  |  |  |
| Mg | 0.58 | 0.39 | 0.57 | 0.35 |
|  | 0.36 | 0.36 | 0.39 | 0.5 |
|  | 0.41 | 0.45 | 0.48 | 0.47 |
|  | 0.45±0.07 | 0.4±0.03 | 0.48±0.05 | 0.44±0.05 |
|  |  |  |  |  |
| Si | 0.05 | 0.17 | 0.31 | 0.13 |
|  | 0.26 | 0.09 | 0.33 | 0.24 |
|  | 0.13 | 0.16 | 0.2 | 0.11 |
|  | 0.15±0.06 | 0.14±0.03 | 0.28±0.04 | 0.16±0.04 |
|  |  |  |  |  |
| P | 1.57 | 1.26 | 1.09 | 1.03 |
|  | 1.59 | 1.4 | 1.1 | 1.13 |
|  | 1.69 | 1.31 | 1.2 | 1.2 |
|  | 1.62±0.04 | 1.32±0.04 | 1.13±0.04 | 1.12±0.05 |
|  |  |  |  |  |
| S | 0.82 | 0.69 | 0.58 | 0.55 |
|  | 1.28 | 0.76 | 0.46 | 0.41 |
|  | 0.87 | 0.8 | 0.39 | 0.5 |
|  | 0.99±0.15 | 0.75±0.03 | 0.48±0.06 | 0.49±0.04 |
|  |  |  |  |  |
| Cl | 0.57 | 0.37 | 0.67 | 0.29 |
|  | 0.39 | 0.29 | 0.49 | 0.44 |
|  | 0.41 | 0.21 | 0.46 | 0.27 |
|  | 0.46±0.06 | 0.29±0.05 | 0.54±0.07 | 0.33±0.05 |
|  |  |  |  |  |
| K | 3.3 | 2.69 | 3.22 | 2.68 |
|  | 3.43 | 2.75 | 3.28 | 2.75 |
|  | 3.29 | 2.8 | 3.49 | 2.67 |
|  | 3.34±0.05 | 2.75±0.03 | 3.33±0.08 | 2.7±0.03 |
|  |  |  |  |  |
| Ca | 0.48 | 0.45 | 0.52 | 0.35 |
|  | 0.29 | 0.49 | 0.64 | 0.49 |
|  | 0.39 | 0.68 | 0.55 | 0.5 |
|  | 0.39±0.05 | 0.54±0.07 | 0.57±0.04 | 0.45±0.05 |
