# Supplementary material for: Elemental and macromolecular modifications in Triticum aestivum L. plantlets under different cultivation conditions
Source: PLoS One. 2018 Aug 28;13(8):e0202441. doi: 10.1371/journal.pone.0202441 (PMC6112624; doi:10.1371/journal.pone.0202441)
Supplement: S3 Table — (DOCX) [file pone.0202441.s003.docx]

Table 3. Raw values of analyses of pigment content in wheatgrass leaves extracts

| **Pigment content (%)** | **Hydroponic/Spring** | **Soil/Spring** | **Hydroponic/Drilling** | **Soil/Drilling** |
| --- | --- | --- | --- | --- |
| **Phaeophytin** | 24.01 | 4.9 | 9.51 | 7.31 |
|  | 23.72 | 4.01 | 9.39 | 7.52 |
|  | 23.02 | 4.03 | 9.37 | 7.4 |
| Mean ± standard error | 23.58±0.29 | 4.31±0.29 | 9.42±0.04 | 7.41±0.06 |
| **Hydroxychlorophyll** a | 64.8 | 39.01 | 61.88 | 24.45 |
|  | 65.07 | 38.83 | 62.14 | 23.99 |
|  | 65.43 | 37.71 | 62.25 | 24.76 |
| Mean ± standard error | 65.1±0.18 | 38.52±0.41 | 62.09±0.11 | 24.4±0.22 |
| **Chlorophyll** a | 52.34 | 41 | 30.07 | 25.79 |
|  | 51.51 | 40.23 | 29.86 | 25.73 |
|  | 52.19 | 40.04 | 30.59 | 26.01 |
| Mean ± standard error | 52.01±0.26 | 40.42±0.29 | 30.17±0.22 | 25.84±0.09 |
