# Supplementary material for: Elemental and macromolecular modifications in Triticum aestivum L. plantlets under different cultivation conditions
Source: PLoS One. 2018 Aug 28;13(8):e0202441. doi: 10.1371/journal.pone.0202441 (PMC6112624; doi:10.1371/journal.pone.0202441)
Supplement: S4 Table — (DOCX) [file pone.0202441.s004.docx]

Table 4. Raw values of analyses of phenolic contents and free radical scavenging activities of wheatgrass juice

|  | Hydroponic - spring | Soil - spring | Hydroponic - drilling | Soil - drilling |
| --- | --- | --- | --- | --- |
| **Total phenolic content (mg gallic acid/ml juice)** | 819.97 | 777.03 | 749.48 | 710.94 |
|  | 809.9 | 778.285 | 779 | 670.45 |
|  | 826.87 | 785.38 | 711.23 | 739.4 |
| Mean ± standard error | 818.91±4.93 | 780.23±2.6 | 746.57±19.62 | 706.93±20 |
| **Total flavonoid content (mg quercetin/ml juice)** | 814.15 | 500.25 | 471.43 | 350 |
|  | 689.75 | 508.87 | 506.2 | 361.54 |
|  | 925.23 | 462.89 | 424.36 | 329.04 |
| Mean ± standard error | 809.71±68.013 | 490.67±14.11 | 467.33±23.71 | 346.86±9.51 |
| **Free radical (DPPH) % scavenging** | 51.19 | 35.04 | 50.02 | 29.01 |
|  | 44.815 | 26.44 | 54.9 | 35.59 |
|  | 50.78 | 53.45 | 41.86 | 25.98 |
| Mean ± standard error | 48.93±2.06 | 38.31±7.97 | 48.93±3.8 | 30.19±2.84 |
